# A landscape analysis of clinical trials in cancer pain management: current trends and emerging therapeutic targets

**DOI:** 10.3389/fonc.2026.1795684

**Published:** 2026-05-19

**Authors:** Jiaqi Cao, Xiaoyi Yu, Wei Lu

**Affiliations:** 1 Anesthesiology, Guizhou Medical University, Guiyang, Guizhou, China; 2 Pain Management Department, Affiliated Hospital of Guizhou Medical University, Guiyang, Guizhou, China

**Keywords:** cancer pain, clinical trials, opioids, precision medicine, trial landscape

## Abstract

**Background:**

Despite a growing volume of clinical research, cancer pain management remains dependent on opioid-centric paradigms with few therapeutic breakthroughs. To identify innovation gaps and guide future research, a systematic evaluation of the global clinical trial landscape is imperative.

**Methods:**

We conducted a comprehensive landscape analysis of 632 interventional cancer pain trials(1994-2025) from the Trialtrove database. Key characteristics including temporal trends, trial phases, therapeutic targets, and primary endpoints were extracted and analyzed.

**Results:**

Our analysis revealed a significant “innovation paradox.” Although trial initiations have increased 3.2-fold since 2000, research remains focused on established mechanisms. Late-stage (Phase III, 38%) investigations predominated over scarce early-phase (Phase I, 13%) studies. Over half (52%) of all trials targeted opioid receptors, while emerging targets like NaV1.7 (18%)—a peripheral sodium channel implicated in inherited pain syndromes—and cannabinoid receptors (5%) were underrepresented. All percentages reflect proportions of the total 632 trials and categories may overlap. Furthermore, primary endpoints overwhelmingly favored unidimensional pain intensity scales (35.8%) over functional or quality-of-life (QoL) metrics. The most advanced innovations are currently limited to dual-mechanism agents (e.g., tapentadol combining MOR agonism and norepinephrine reuptake inhibition) and novel delivery systems (e.g., iontophoretic transdermal patches or intrathecal pumps).

**Conclusion:**

The cancer pain research ecosystem exhibits substantial volume growth unmatched by mechanistic innovation. To surmount this therapeutic stagnation, future priorities must include expanding early-phase trials on novel pathways (e.g., tumor-microenvironment interactions), adopting composite endpoints integrating pain with function and QoL, and leveraging real-world evidence and AI-driven phenotyping to advance precision analgesia. This paradigm shift is critical to addressing the multidimensional burden of cancer pain.

## 1 Introduction

### 1.1 The global burden of malignancy-associated pain

Malignancy-associated pain constitutes one of the most pervasive and distressing sequelae of oncologic disease and affects a substantial portion of the global patient population. Data from recent epidemiological meta-analyses indicate a prevalence of approximately 39–44% following curative treatment, which escalates to over 55% during active anticancer therapy and reaches as high as 66–80% in patients with advanced, metastatic, or terminal illness (1, 2). Despite advancements in palliative care, pain remains inadequately controlled in nearly one-third of cases, thereby contributing to a phenomenon often described as “total pain,” a multidimensional burden that encompasses physical suffering, psychological distress, functional impairment, and diminished quality of life (3). The escalating global cancer incidence, driven by aging demographics and improved survival rates, suggests that the absolute number of patients requiring complex analgesic management will continue to surge and consequently pose a critical challenge to healthcare systems worldwide.

### 1.2 Limitations of the current therapeutic paradigm

The cornerstone of current management strategies remains the World Health Organization (WHO) analgesic ladder introduced in 1986, which is a framework that advocates for a stepwise escalation from non-steroidal anti-inflammatory drugs (NSAIDs) to weak opioids and subsequently to strong opioids (4). While this algorithmic approach has provided a standardized foundation for clinical care, its limitations are increasingly apparent given the heterogeneous nature of modern oncologic pain. A primary limitation is its reliance on non-specific systemic analgesia, a method that often fails to effectively manage breakthrough cancer pain (BTcP). Breakthrough cancer pain involves transient exacerbations of severe pain occurring against a background of controlled baseline pain and affects 40–80% of patients (5). Furthermore, this “opioid-centric” model is fraught with numerous complications, including tolerance, opioid-induced hyperalgesia (OIH), respiratory depression, and gastrointestinal toxicity. The dual crisis of opioid misuse and the resultant regulatory tightening has further complicated prescribing practices and paradoxically led to the undertreatment of legitimate medical needs (4).

### 1.3 Pathophysiological complexity: beyond nociception

The current therapeutic stagnation is partly attributable to a historical oversimplification of cancer pain mechanisms. Contemporary neuroscience has reframed this condition not merely as a consequence of tissue injury or compression but as a dynamic pathological state driven by the tumor microenvironment (TME) (6). Malignant cells engage in complex bidirectional crosstalk with both the peripheral and central nervous systems. Within the TME, the secretion of pronociceptive mediators such as nerve growth factor (NGF), endothelin-1, glutamate, and pro-inflammatory cytokines like TNF-α and IL-6 induces peripheral sensitization and neuroinflammation. This chemical milieu thereby facilitates the transition from acute nociception to chronic neuropathic states characterized by central sensitization and structural neuroplasticity (7). Consequently, traditional opioids often lack efficacy against such mixed-mechanism pain syndromes, thus highlighting the necessity for a strategic shift towards mechanism-based treatments that target specific molecular pathways rather than broad symptom suppression.

### 1.4 The rationale for a landscape analysis

Given the disconnect between the biological complexity of neoplastic pain and the limitations of the existing pharmacopoeia, clinical trials serve as the critical bridge for translating mechanistic insights into therapeutic innovation. A systematic evaluation of the global research ecosystem is lacking, however, and it remains unclear whether the current investigational pipeline adequately addresses the need for non-opioid targets, precision medicine approaches, and novel delivery systems. This study therefore aims to conduct a comprehensive landscape analysis of interventional clinical trials registered over the past three decades. By mapping temporal trends, geographical distribution, and the evolution of therapeutic targets, we seek to identify innovation gaps and define strategic priorities to ultimately break the current impasse in cancer pain management.

## 2 Materials and methods

### 2.1 Data source

The clinical trial data for this analysis were procured from the Trialtrove database, a leading clinical intelligence platform, with all searches concluded by December 2025. This database aggregates information from major international registries, including ClinicalTrials.gov, the EU Clinical Trials Register, and the national registries of China and Japan. Its comprehensive scope and structured data curation have underpinned numerous high-impact publications in both oncology and pain research, thereby affirming its robustness as a source for competitive landscape assessment.

### 2.2 Search strategy

A systematic search was performed to identify interventional clinical trials focused primarily or secondarily on cancer pain or nociceptive pain associated with malignancy. The search strategy utilized a combination of terms such as “cancer pain,” “oncologic pain,” “pain, neoplastic,” and “malignancy-associated pain,” along with “nociceptive pain” paired with either “cancer” or “tumor.” This search was designed to encompass all trial phases and statuses registered up to the specified end date.

### 2.3 Data extraction and analysis

Two reviewers independently screened the results and extracted relevant data pertaining to trial volume and temporal trends from 2000 to 2025, geographical distribution, clinical trial phase (I–IV), the nature of therapeutic interventions (pharmacological versus non-pharmacological), trial status, and specified molecular targets. The extracted dataset was subsequently cleaned and analyzed utilizing Microsoft Excel and R (version 4.3.1), from which descriptive statistics and visualizations were generated.

### 2.4 Quality assurance and limitations

To ensure data integrity and minimize extraction bias, a process of cross-verification was implemented wherein a random 10% sample of the dataset was reviewed by both researchers, with any discrepancies resolved through consensus, and unstructured fields were subjected to manual curation. The limitations of this study encompass potential classification biases inherent to the database, regional variations in the completeness of data, and the exclusion of preclinical studies not registered in public databases. The large-scale nature of the dataset, however, ensures the validity of the overarching trends identified herein.

## 3 Results

Our search identified 632 interventional clinical trials that were registered between 1994 and 2025.

### 3.1 Temporal trends and geographical landscape

**Figure 1:**
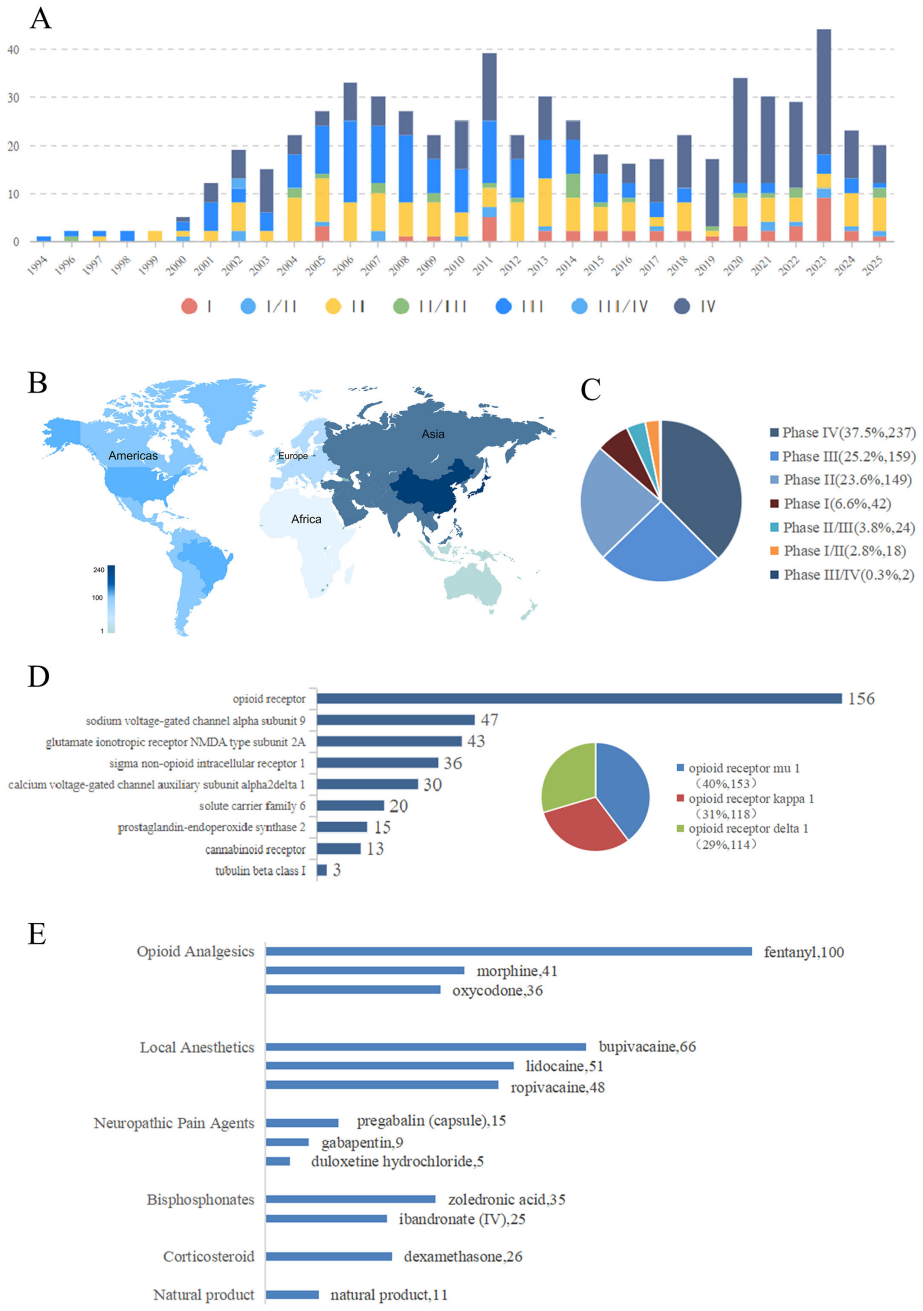
**(A)** Provided a comprehensive analysis of global cancer pain clinical trials by combining the year, clinical trial phases, and the number of clinical trials. **(B)** Analyzed global cancer pain clinical trials from the perspective of national distribution. **(C)** Analyzed global cancer pain clinical trials from the clinical trial phases. **(D)** Analyze the main targets of clinical drug therapy for cancer pain. The inset in the lower right corner shows the proportion of different opioid receptors in the experiment. **(E)** Analyze the main drugs in the clinical treatment of cancer pain.

Trial activity has exhibited a consistent upward trajectory over the past three decades and since 2002, the annual volume of trial launches has shown an accelerating though fluctuating growth pattern, culminating in a 3.2-fold increase. This trend included distinct peaks in activity observed in 2006, 2011, and 2023, a surge that correlates with the global prioritization of palliative care and the diversification of analgesic modalities (**Figure 1A**). Geographically, the research landscape has become increasingly polarized. Asia, driven primarily by China and Japan, accounts for 38% of trials and North America, with the United States being predominant, contributes 23%, thereby these two regions collectively represent 61% of global activity. Europe contributes 16% and the remainder is distributed across other regions. This geographical distribution reflects a complex interplay between regional disease burden and varying regulatory frameworks. Notably, China’s recent integration into the International Council for Harmonization (ICH) has catalyzed a surge in high-quality, multicenter trials and has strengthened its collaboration with international institutions (8). Conversely, the rigorous regulatory environment in the USA continues to anchor high-data-quality investigations, particularly for novel entities, even as a gradual shift of late-stage trials toward emerging markets for cost-efficiency is observed (**Figure 1B**).

### 3.2 Clinical development phase distribution

The distribution across trial phases reveals a “maturity bias” within the current development pipeline. Phase III trials represent the largest proportion at 38% and are followed by Phase IV post-marketing surveillance at 25% and Phase II studies at 24%. In stark contrast, Phase I trials account for merely 13% of the total. This relatively low proportion of early-phase investigations underscores a scarcity of first-in-human studies for novel mechanisms. The dominance of late-stage trials thus suggests a strategic focus on the reformulation and label expansion of existing molecules rather than on *de novo* drug discovery, a trend that could potentially limit the introduction of breakthrough therapies possessing fundamentally new mechanisms of action (9) (**Figure 1C**).

### 3.3 Therapeutic target analysis

An analysis of molecular targets unveiled a distinct and persistent hierarchy. The opioid receptor family remains the monolithic focus and encompasses 52% of all trials. Within this opioid-centric subset, classical μ-opioid receptor (MOR) agonists constitute 40% of investigations and are followed by κ-opioid (KOR) at 31% and δ-opioid (DOR) receptor agonists at 29%. This distribution highlights a continued industry effort to engineer “biased ligands” or alternative opioid subtypes with the potential to dissociate analgesia from adverse effects such as respiratory depression and abuse liability. Among non-opioid targets, the voltage-gated sodium channel NaV1.7 has emerged as the most significant novel target at 18%, which reflects intense interest in selective peripheral blockers for neuropathic pain (10). Clinical translation has, however, been hindered by selectivity challenges and off-target effects. Other notable targets aimed at central sensitization include NMDA receptor subunits at 13% and calcium channel subunits at 10%. Less frequent yet emerging targets include the cannabinoid system at 5% and serotonin transporters at 2%. A “miscellaneous” category of targets, which comprises 10% of trials and includes TRP channels and pro-inflammatory cytokines, indicates a fragmented but growing interest in targeting the inflammatory milieu of the tumor microenvironment (**Figure 1D**).

### 3.4 Pharmacological interventions

The pharmacological landscape largely mirrors current clinical practice standards. Among opioids, fentanyl is the most prevalent investigational agent at 15.8%, with a particular focus on innovative transdermal and sublingual delivery systems, and is followed by morphine at 6.5% and oxycodone at 5.7%. This trend underscores an ongoing optimization of “gold standard” opioids rather than their replacement. Local anesthetics such as bupivacaine at 10.4% and lidocaine at 8.1% maintain their prominence, a fact largely driven by advances in regional anesthesia and interventional techniques. Adjuvants for neuropathic pain, including pregabalin at 2.4% and gabapentin at 1.4%, show a stable yet limited trial volume. Bisphosphonates like zoledronic acid at 5.5% are frequently tested for bone metastasis pain specifically, while corticosteroids such as dexamethasone at 4.1% are investigated for their dual anti-inflammatory and anti-emetic benefits. Notably, natural products comprise only 1.7% of all interventions, suggesting that they remain on the periphery of mainstream clinical development despite their widespread patient usage (**Figure 1E**).

### 3.5 Trial status and endpoint utilization

**Figure 2:**
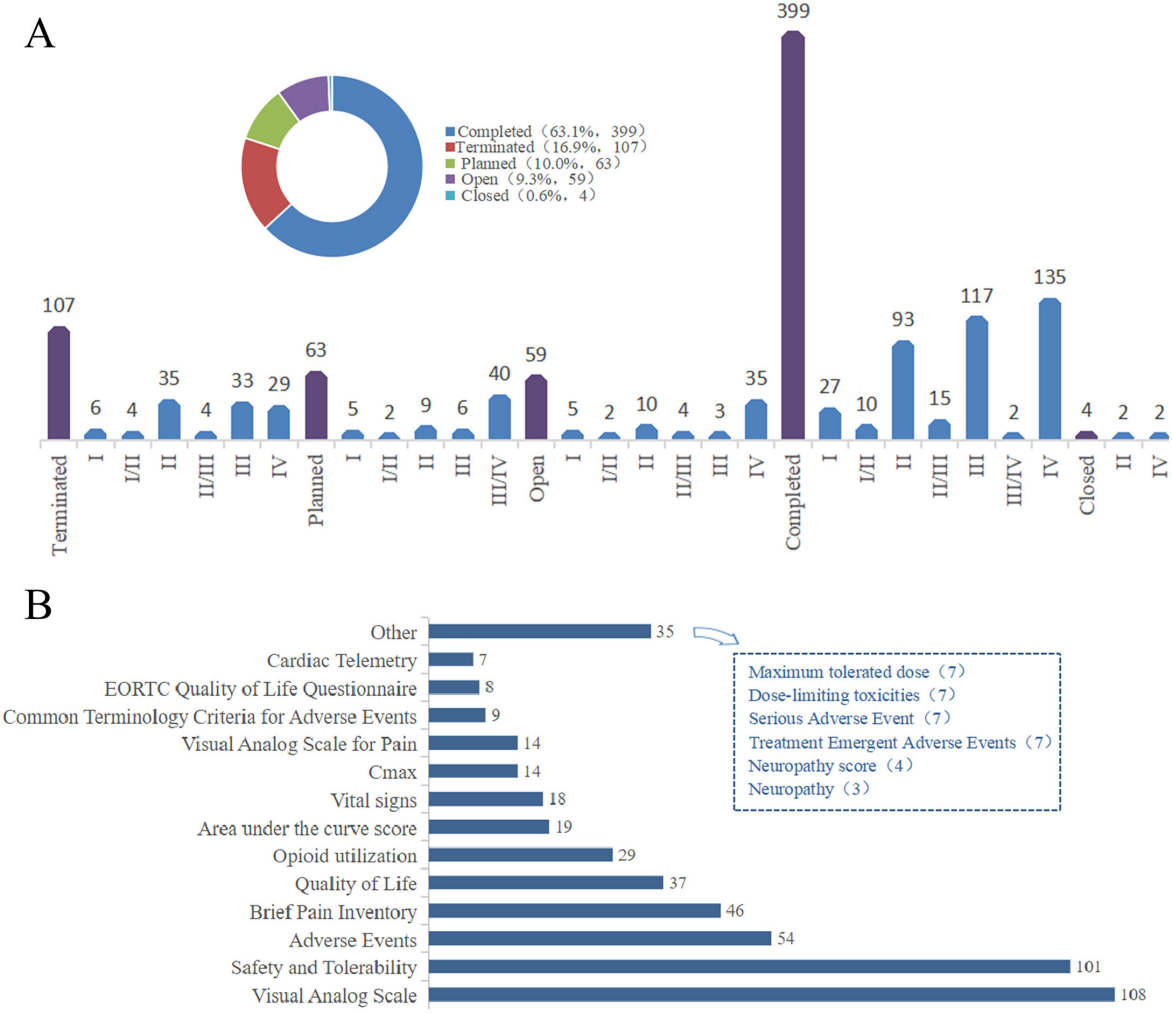
**(A)** Analyzed the distribution of cancer pain treatment modalities based on the stages of clinical trials and the proportion of each phase within those stages. **(B)** Analyzed the primary endpoint group of clinical treatment of cancer pain.

Regarding trial status, 63.1% of studies have been completed, whereas a concerning 16.9% were terminated prematurely. The primary reasons cited for early termination are recruitment failures and funding withdrawal, which highlights the logistical challenges inherent in conducting trials within a fragile palliative population. Active or recruiting trials currently constitute 9.3% of the dataset (**Figure 2A**).

Endpoint selection analysis reveals a heavy reliance on subjective, unidimensional metrics. Pain intensity scales such as the NRS and VAS dominate as the primary endpoint and collectively account for 35.8% of trials, while safety and tolerability assessments represent another 25.5%. Crucially, multidimensional assessment tools like the Brief Pain Inventory (BPI), which captures the interference of pain with daily function, are utilized as the primary endpoint in only 7.6% of trials. Even more strikingly, Quality of Life (QoL) measures serve as the primary endpoint in just 6.1% of studies. This disparity suggests a lingering disconnect between regulatory approval criteria that are often based on simple pain reduction and patient-centered outcomes that focus on functional recovery and overall life quality (**Figure 2B**).

## 4 Discussion

### 4.1 The innovation paradox in cancer pain research

A striking disconnect persists between the burgeoning volume of clinical trials and the stagnant pace of transformative therapeutic innovation, a phenomenon we have termed the “**innovation paradox**”: a >3-fold increase in trial volume since 2000 juxtaposed with over half of all investigations (52%) still anchored to opioid pathways and a mere 13% dedicated to first-in-human Phase I studies. This persistence is notable given the well-recognized limitations of chronic opioid therapy—including opioid-induced hyperalgesia, tolerance, and endocrine effects—although opioids remain indispensable for moderate-to-severe cancer pain in many clinical scenarios (11). A predominance of Phase III trials at 38% and Phase IV trials at 25%, contrasted with the paucity of Phase I investigations at only 13%, suggests a risk-averse industry strategy that, while understandable given the high regulatory hurdles for first-in-class analgesics and the challenges of demonstrating superior efficacy over established opioids, may inadvertently limit mechanistic diversity. This strategy appears to favor the reformulation of existing agents into imitative drugs to ensure regulatory success instead of pioneering novel mechanisms of action (9). Furthermore, although Asia’s contribution to the global trial landscape is surging to 38%, a substantial proportion of these studies appear to replicate Western protocols involving generic opioids (based on an exploratory analysis of intervention types and primary endpoints; formal quantitative assessment of protocol originality was beyond the scope of this study). It is important to note that such replication studies serve a valid purpose in establishing regional safety and efficacy profiles. However, this pattern also represents a missed opportunity to explore culturally distinct pain phenotypes or to rigorously evaluate integrative medicine approaches that could offer significant opioid-sparing benefits.

To leverage Asia’s trial infrastructure for genuine innovation, future efforts could: (1) systematically validate culturally specific pain phenotypes (e.g., higher reported neuropathic components in certain Asian populations) using validated cross-cultural instruments; and (2) incorporate rigorously designed integrative medicine arms—such as standardized acupuncture or herbal formulas (e.g., Corydalis yanhusuo extracts) with known opioid-sparing potential—in pragmatic platform trials, provided that active comparators and sham controls are employed to minimize bias (12).

### 4.2 Emergent therapeutics: from bench to bedside

The development of dual-action molecules such as tapentadol, which is a MOR agonist and norepinephrine reuptake inhibitor, and oxycodone/naloxone fixed-dose combinations represents a rational evolution in polypharmacology. These agents have demonstrated analgesia comparable to pure mu-agonists and possess improved tolerability profiles, particularly concerning bowel function (13). They remain, however, fundamentally opioid-centric and thus fail to fully address the complex, non-opioid-responsive neuropathic components of cancer pain.

Technological advances like intrathecal drug delivery systems (IDDS) have demonstrated superior efficacy and infection control when compared to external catheters and allow for significant systemic dose reductions which in turn minimizes toxicity. Despite high-level evidence supporting their cost-effectiveness and utility in refractory pain, IDDS trials comprise only 3.2% of the current pipeline (14). This underutilization is likely attributable to high initial implementation costs combined with the requirement for specialized surgical training and a lack of sufficient reimbursement pathways in many healthcare systems.

The quest for non-opioid targets concurrently faces significant translational hurdles. For instance, voltage-gated sodium channel NaV1.7 inhibitors showed immense promise in preclinical models and genetic studies but have largely failed to demonstrate robust efficacy in clinical trials, a failure stemming from issues with isoform selectivity and central target engagement (15). The cannabinoid system similarly remains a controversial frontier. Strong preclinical rationale for the use of cannabinoids in neuropathic pain is contrasted by recent systematic reviews indicating that current formulations often lack sufficient high-quality evidence for their recommendation as standard analgesics, thereby highlighting a critical need for better-designed trials (16).

Collectively, these examples underscore a central tension of the innovation paradox: while transformative non-opioid targets (NaV1.7, cannabinoids) offer the prospect of mechanism-based analgesia, their high attrition rates discourage early-phase investment, perpetuating a cycle where incremental refinements of existing opioids dominate the pipeline—a cycle that will likely persist without new funding models or regulatory incentives for first-in-class pain therapeutics.

### 4.3 Future research directions

#### 4.3.1 Targeting the tumor microenvironment–neuroimmune axis

Recent advances in cancer neuroscience have illuminated the nervous system as an active, dynamic regulator of the tumor microenvironment (TME) rather than a passive bystander. Bidirectional crosstalk between tumor cells, immune cells, and peripheral nerves drives both cancer progression and nociceptive sensitization (17). Specific Recommendation: Future research should prioritize the rational design and development of monoclonal antibody-based therapeutics that selectively target the CCL2-CCR2 chemokine axis and the IL-6-gp130 cytokine signaling pathway. Mechanistically, cancer-induced nerve injury (CINI) triggers neurons to initiate an IL-6-mediated inflammatory response that fosters a globally immunosuppressive TME, while activated Schwann cells and sensory neurons utilize CCL2 to recruit tumor-associated macrophages, ultimately exacerbating perineural invasion and pain (18).

#### 4.3.2 Implementing adaptive platform trials

To overcome the “innovation paradox” and the maturity bias currently plaguing late-stage pain trials, clinical investigations must transition away from traditional, rigid trial structures. Specific Recommendation: We strongly recommend establishing master protocol-driven umbrella and platform trials to concurrently evaluate multiple investigational agents or combinatorial regimens against a common, molecularly defined tumor cohort. As highlighted by Park et al. (19), master protocols offer enhanced efficiency by sharing operational infrastructure across multiple sub-studies. Platform trials, in particular, provide a perpetual testing environment with pre-specified adaptation rules that allow researchers to drop ineffective intervention arms and introduce new ones without halting the trial (19, 20).

#### 4.3.3 Developing digital biomarkers for pain phenotyping

The current reliance on subjective, unidimensional pain intensity scales (e.g., NRS, VAS) represents a critical methodological flaw in capturing the multidimensional nature of “total pain.” Specific Recommendation: To advance precision analgesia, there is an urgent need to engineer non-invasive digital biomarker panels for real-time cancer pain phenotyping. This can be achieved by leveraging physiological data, such as electrocardiogram (ECG) and electrodermal activity (EDA), captured continuously via wearable biosensors (21).

The application of machine learning (ML) and deep learning (DL) algorithms to these high-dimensional datasets enables the development of predictive models capable of identifying patterns that precede breakthrough cancer pain (BTcP) events, potentially allowing for pre-emptive intervention (22). Furthermore, the combination of digital phenotyping with pharmacogenomic data can help stratify patients based on their likelihood of response or risk of opioid-induced toxicity. To ensure the generalizability and privacy of such models, future research should adopt federated learning frameworks, which allow algorithms to be trained across multiple decentralized institutions without the need to share sensitive patient data (23). The incorporation of these composite, patient-centric endpoints—integrating continuous physiological monitoring with functional outcomes like those captured by the Brief Pain Inventory (BPI)—will be essential for regulatory approval of next-generation analgesics and for truly capturing the value of a palliative intervention (24).
